# State of the art of urine treatment technologies: A critical review.

**DOI:** 10.1016/j.wroa.2021.100114

**Published:** 2021-08-19

**Authors:** Tove A. Larsen, Michel E. Riechmann, Kai M. Udert

**Affiliations:** 1 Eawag, Swiss Federal Institute of Aquatic Science and Technology, 8600 Dübendorf, Switzerland; 2 ETH Zürich, Institute of Environmental Engineering, 8093 Zürich, Switzerland

**Keywords:** Nitrogen Recovery, Phosphorus Recovery, Pharmaceutical Removal, Environmental Protection, Volume Reduction, Energy Production

## Abstract

Over the last 15 years, urine treatment technologies have developed from lab studies of a few pioneers to an interesting innovation, attracting attention from a growing number of process engineers. In this broad review, we present literature from more than a decade on biological, physical-chemical and electrochemical urine treatment processes. Like in the first review on urine treatment from 2006, we categorize the technologies according to the following objectives: stabilization, volume reduction, targeted N-recovery, targeted P-recovery, nutrient removal, sanitization, and handling of organic micropollutants. We add energy recovery as a new objective, because extensive work has been done on electrochemical energy harvesting, especially with bio-electrochemical systems. Our review reveals that biological processes are a good choice for urine stabilization. They have the advantage of little demand for chemicals and energy. Due to instabilities, however, they are not suited for bathroom applications and they cannot provide the desired volume reduction on their own. A number of physical-chemical treatment technologies are applicable at bathroom scale and can provide the necessary volume reduction, but only with a steady supply of chemicals and often with high demand for energy and maintenance. Electrochemical processes is a recent, but rapidly growing field, which could give rise to exciting technologies at bathroom scale, although energy production might only be interesting for niche applications. The review includes a qualitative assessment of all unit processes. A quantitative comparison of treatment performance was not the goal of the study and could anyway only be done for complete treatment trains. An important next step in urine technology research and development will be the combination of unit processes to set up and test robust treatment trains. We hope that the present review will help guide these efforts to accelerate the development towards a mature technology with pilot scale and eventually full-scale implementations.

## HIGHLIGHTS

· The last 15 years, we have seen a tremendous development in urine treatment technologies
· We discuss relevant technologies for reaching eight process-engineering goals (sections 2-8)
· A few biological as well as physical-chemical technologies have been piloted and are moving towards industrialization
· There is a boost of experimental electrochemical treatment technologies, which hold promise for on-site installations

## Graphical Abstract

**Figure:**
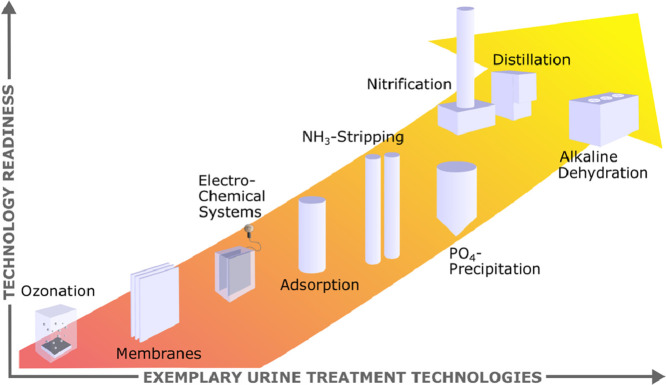

**Abbreviations**
AEM: Anion Exchange Membrane
AOB: Ammonia Oxidizing Bacteria
AOP: Advanced Oxidation Process
BDD: Boron-Doped Diamond electrode
β_S_: Super-saturation, defined as IAP/K_SP_, where IAP is the ion activation product, and K_SP_ the thermodynamic equilibrium constant
CBP: Chlorination By-Product
CEM: Cation Exchange Membrane
COD: Organic matter expressed as Chemical Oxygen Demand
CSTR: Continuous flow Stirred Tank Reactor
DCMD: Direct Contact Membrane Distillation
EC: Electrolysis Cell
ED: ElectroDialysis
FBR: Fluidized Bed Reactor
FC: Fuel Cell
FO: Forward Osmosis
HRT: Hydraulic Retention Time
LDH: Layered Double Hydroxides
MABR: Membrane-Aerated Biofilm Reactor
MAP: Magnesium Ammonium Phosphate
MBR: Membrane BioReactor
MD: Membrane Distillation
MEC: Microbial Electrolysis Cell
MFC: Microbial Fuel Cell
MPP: Magnesium Potassium Phosphate
NF: Nano Filtration
NH_tot_: Total ammonia nitrogen (= NH_4_^+^ + NH_3_)
NOB: Nitrite Oxidizing Bacteria
PBBR: Packed Bed Biofilm Reactor
PDS: PeroxyDiSulfate
SBR: Sequencing Batch Reactor
SHE: Standard Hydrogen Electrode
SI: Saturation Index (SI = log_10_ (β_S_)
TDIROF: Thermally Decomposed IrO_2_ Film anode
TOC: Total Organic Carbon
UV: UltraViolet radiation
WWTP: WasteWater Treatment Plant

## 1 Introduction

The community of wastewater professionals has worked on the concept of urine source separation since the mid 1990’ties, with the double purpose of water pollution control and recycling of nutrients, mainly for agriculture. In an early critical review of potential urine treatment technologies, Maurer et al. (2006) defined seven process-engineering objectives to reach the double purpose of water pollution control and reuse of nutrients in agriculture: stabilization, volume reduction, targeted N-recovery, targeted P-recovery, nutrient removal, sanitization, and handling of organic micropollutants. In the present review, we take up this structure, adding the goal of energy recovery.

The rationale of urine source separation for improved water pollution control is based on the fact that urine provides most of the nitrogen and phosphorus contained in household wastewater (Larsen and Gujer 1996). The large emissions of nitrogen and phosphorus are considered some of the most critical global environmental challenges, with a high risk of destabilizing the Earth's ecosystem, mainly due to eutrophication in fresh water ecosystems and coastal areas (Steffen et al. 2015). Separate collection and treatment of urine, combined with small compact wastewater treatment plants (WWTPs) for the remaining wastewater, removes nutrients as efficiently as the most advanced WWTPs for mixed wastewater known today (Wilsenach and van Loosdrecht 2006). At a global scale, this is important because only around 10 % of the world's population has access to nutrient-eliminating WWTPs (Larsen 2011). Further, about two thirds of the pharmaceuticals excreted by the human metabolism and about half of the corresponding eco-toxicological potential of those pharmaceuticals are contained in urine (Lienert et al. 2007a, Lienert et al. 2007b). With increasing concern about organic micropollutants in aquatic ecosystems (Eggen et al. 2014), there would be benefits from removing pharmaceuticals from the aquatic ecosystems through urine separation.

Nutrient recovery from urine can simplify the recycling of nutrients from the human metabolism to agriculture. In the case of phosphorus, food security as well as the environment would strongly benefit from recycling strategies because of the increasing economic and environmental costs of phosphorus mining (Cordell and Neset 2014). In the case of nitrogen, a freely available resource from the atmosphere, there are three main arguments for recycling from urine: energy demand, economic value, and reducing the emissions of hazardous nitrogen compounds to the environment. Removal of nitrogen from wastewater and production of nitrogen fertilizer are both energy-intensive processes (Maurer et al. 2003). Dependent upon technologies, recycling of nitrogen from urine can be more energy-efficient than removal from wastewater and subsequent industrial fertilizer production. Economically, nitrogen is by far the most valuable nutrient in urine (Etter et al. 2011). Where fertilizers are expensive or even unavailable for farmers, especially in low-income countries, urine may be a cost-effective substitute (Andersson et al. 2013). Many recycling technologies provide additional nutrients like phosphorus, potassium and sulfur with high importance for the quality of the crops (Jönsson and Vinneras 2013). On WWTPs, nitrous oxide emissions have recently proven much more important than expected, often surpassing the climate effect of CO_2_ emissions from electricity demand at the plants (Gruber et al. 2020). As pointed out by Steffen et al. (2015), curbing the production of reactive nitrogen and the associated production of climate gases during the entire lifetime of the compound is one of the most important goals of the 21^st^ Century. With a global excretion of around 30 MT·yr^−1^ of nitrogen in urine (roughly 10·g·p^−1^·d^−1^), a recycling strategy could contribute significantly to reducing the requirements for industrially produced reactive nitrogen, which is at present around 120 MT·yr^−1^ (Razon 2018).

The quality of a recycled product from urine must align with the requirements of the customers, in most cases farmers. Besides fertilizer value, hygiene and stability are therefore important factors defining quality. Although the removal of organic micropollutants is disputed based on the comparison with animal manure, which equally contains organic micropollutants (Hammer and Clemens 2007), at least in Switzerland, it is required for urine-based fertilizers intended for edible crops (www.vuna.ch/aurin).

Since the first overview article on urine treatment technologies was published by Maurer et al. (2006), more targeted review articles have been published. However, none of them provides a structured overview of the available technologies to reach the treatment goals of urine source separation. A number of reviews have occurred with a focus on implementation in low-income countries (Rahman et al. 2015, Simha and Ganesapillai 2017), and more specific reviews have been published on selected technologies for urine treatment. Kabdaşlı and Tünay (2018) focus on struvite precipitation, ion exchange and adsorption; Patel et al. (2020) give a detailed overview of membrane and electrochemical technologies, and finally, Chipako and Randall (2020) present technologies optimizing nutrient recovery, with a focus on the pH-dependence of these technologies. For more in-depth discussions than we are able to provide in this comprehensive review, we refer to these specific reviews.

In a recent paper, Larsen et al. (2021) discuss the requirements for global diffusion of the radical innovation of urine source separation. The two most important technical elements defined are (1) mass-produced urine-separating toilets adapted to the socio-economic environment and (2) mass-produced treatment technology. While a new toilet technology for high-income countries entered the market already and several versions are in development for low-income countries, this present review discusses the state of the art of treatment technology development. For the complex socio-economic aspects of global diffusion, we refer to Larsen et al. (2021).

## 2 Composition of fresh and stored urine

There is a huge difference between the properties of fresh and stored urine. Whereas fresh urine contains a high concentration of urea, this compound is rapidly hydrolyzed to ammonia and carbon dioxide as soon as urine enters the non-sterile environment, leading to the release of ammonia and bicarbonate and causing a pH increase (Udert et al. 2006).

(Equation 1) $$H_{2}N\left(CO\right)NH_{2}+2H_{2}O\to NH_{3}+N{H_{4}}^{+}+HC{O_{3}}^{-}$$

The most relevant compounds in urine are nutrients and organic micropollutants, with concentrations in fresh urine mainly depending on diet and medication, respectively. The concentration of heavy metals is so low that it is not an issue (Ronteltap et al. 2007a), but due to cross-contamination from faeces, source-separated urine can contain pathogens (Bischel et al. 2015a, Höglund et al. 1998). The pH increase observed in stored urine leads to precipitation processes altering the concentration of a number of ions, most importantly a substantial decrease in phosphorus concentration of around 30% and a nearly complete removal of calcium and magnesium ions (Udert et al. 2003). These precipitation processes depend on the composition of fresh urine, but also on the amount and composition of co-separated flush water, especially the calcium and magnesium content. During urine collection and storage in ventilated rising pipes and tanks, volatilization of ammonia can lead to substantial nitrogen loss (Siegrist et al. 2013). In Table 1, we list values for pH, electrical conductivity, and concentrations in undiluted fresh and stored urine. Please note that in practice, source-separated urine is often diluted with some flushing water. On average, the production of urine is 1.4 L·p^−1^·d^−1^ (DWA 2016).

**Table 1:** Key components in urine: concentration ranges for fresh urine and typical values for stored urine

	Unit	Fresh urine	Stored urine
pH	-	5.5–7.0^1^	9.1^2^
Electrical conductivity	mS·cm^−1^	8.7–31^3^	30^4^
Organic matter (COD)	mg_O2_·L^−1^	6,300–18,000^1^	10,000^2^
Total nitrogen	mg_N_·L^−1^	4,000–14,000^1^	9,200^2^
Urea	mg_N_·L^−1^	3,400–12,000^1^	0
Total carbonate	mg_C_·L^−1^	0^2^	3,200^2^
Total ammonia nitrogen, NH_tot_ (= NH_4_^+^ + NH_3_)	mg_N_·L^−1^	130–730^1^	8,100^2^
Total phosphorus	mg_P_·L^−1^	350–2,500^1^	540^2^
Potassium	mg_K_·L^−1^	750–2,600^1^	2,200^2^
Total sulfur	mg_S_·L^−1^	600–1,300^5^	Similar to fresh urine
Chloride	mg_Cl_·L^−1^	2,300–7,700^2^	3,800^2^
Sodium	mg_Na_·L^−1^	1,800–5,800^2^	2,600^2^
Magnesium	mg_Mg_·L^−1^	70–120^1^	0^2^
Calcium	mg_Ca_·L^−1^	32–230^1^	0^2^
Single pharmaceuticals	μg·L^−1^	1–1,000^6^	Similar to fresh urine 7
Total estrogens (women's urine)	μmol·L^−1^	0.053–0.27^8^	Data not available 9
Alkalinity	meq·L^−1^	22^2^	490^2^

1 Rose et al. (2015); range based on literature review; urea range calculated as 85% of TN according to Udert et al. (2006)

2 Udert et al. (2006); values for fresh urine taken from literature. Values for stored urine calculated from values for fresh urine by assuming known transformation processes in stored urine.

3 Friedler et al. (2013); range based on literature review

4 Grau et al. (2015); blended stored urine from various collection tanks in Durban (South Africa)

5 DWA (2016); range based on literature review

6 Bischel et al. (2015a); range of average values for 12 different pharmaceuticals based on theoretical calculations

7 Hardly any degradation of pharmaceuticals during storage, according to Ozel Duygan et al. (2021)

8 Xu et al. (1999); range of average values, variation according to the menstrual cycle. Assuming 1.4 L_urine_·p^−1^·d^−1^

9 There is some evidence that estrogens may be partially degraded during storage (Arias et al. 2019)

## 3 Stabilization of urine

Following Maurer et al. (2006), urine stabilization includes processes, which (i) degrade organic matter, thus preventing malodor, (ii) prevent volatilization of NH_3_ and (iii) prevent unwanted precipitation, which can result in operational problems such as pipe clogging or membrane fouling.

### 3.1 Biological processes for stabilization

Biological stabilization primarily aims at reducing the pH-value of urine through nitrification and fulfills the goal of removing most of the organic matter, including malodorous compounds (Udert and Wächter 2012). A biological process with its high enzymatic activity will rapidly convert any remaining urea to ammonium (Coppens et al. 2016), and it thus makes no difference whether the substrate is fresh or stored urine. However, the nitrifying community will differ, because not all nitrifying bacteria produce urease (De Paepe et al. 2018).

Partial nitrification is the most frequent biological stabilization method, but a number of authors used complete nitrification for the provision of a stabilized substrate for algae growth (Coppens et al. (2016), Feng et al. (2008); section 4.4), or for the provision of a stabilized substrate for volume reduction by electrodialysis (ED; De Paepe et al. (2018); section 4.3). Although ammonium and nitrite oxidation are normally unproblematic in conventional wastewater treatment, high concentrations of salt (Moussa et al. 2006), free ammonia and nitrous acid (Anthonisen et al. 1976) may limit the reaction rates in concentrated urine solutions and can lead to an imbalance between ammonium-oxidizing bacteria (AOB) and nitrite oxidizing bacteria (NOB) (Udert and Wächter 2012).

#### 3.1.1 Partial nitrification of urine: Biological production of ammonium nitrate

Based on the alkalinity in urine only, mainly originating from urea hydrolysis (Equation 1), nitrification results in approximately equal amounts of total ammonia (NH_tot_) and nitrate (Udert and Wächter 2012). During ammonia oxidation, pH decreases to a value of around 5.4, at which the activity of *Nitrosomonas eutropha*, the dominant AOB in the process, ceases due to energy limitations (Fumasoli et al. 2015). Keeping pH in a narrow range, e.g. by controlling the inflow rate of urine (Udert and Wächter 2012), is the key to stable nitrate production. Otherwise, partial nitrification may result in ammonium nitrite (section 6.1.1).

According to the literature until 2019, stable partial nitrification has only been achieved in biofilm reactors (Table 2), but at similar nitrification rates per surface as in municipal wastewater systems: Sørensen and Morgenroth (2020) cite values between 0.5 and 2.5 g_N_·m^−2^·d^−1^. In the early works on urine nitrification, emphasis was not on optimization, but on feasibility (Feng et al. 2008) and stability of the process (Udert and Wächter 2012), with the latter process running stable for 12 months. In a long-running pilot plant, Fumasoli et al. (2016) found that the nitrification rate was inversely correlated with the inlet NH_tot_ concentration (Table 2), showing that the inhibition by salt and/or free ammonia is in fact relevant for urine nitrification. Based on typical daily nitrogen excretions in urine (Table 1), this results in a reactor size of around 50 L·p^−1^ for the highly concentrated urine, and around 10 L·p^−1^ for the more diluted urine, with a trade-off between reactor size for partial nitrification and energy demand of subsequent evaporation processes (section 4.1). The major challenge for the stability of partial nitrification is inhibition of NOB by nitrous acid, caused by accumulation of nitrite (Fumasoli et al. 2016). Another challenge is the growth of acid-tolerant AOB, when urine nitrification is operated at low pH values. If acid-tolerant AOB are allowed to grow in, they may cause a pH decrease to values as low as 2.2, resulting in emission of large amounts of hazardous volatile nitrogen compounds such as NO, N_2_O, NO_2_ and HNO_2_ with detrimental effects on air quality and climate, and a loss of acid-sensitive nitrifiers (Fumasoli et al. 2017). While it is easy to keep pH within narrow bands, there is a lack of methods to monitor and control the nitrite concentration (Mašić et al. 2015). An additional research gap exists with respect to the emission of nitrous oxides during normal operation, a problem that has gained increased importance for conventional wastewater treatment (Gruber et al. 2020).

**Table 2:** Results for partial and full nitrification

Reactor	pH	T	Inlet C_NH,tot_	Volumetric rate	Surface rate	Reference
	[-]	[°C]	[g_N_·m^−3^]	[g_N_·m^−3^·d^−1^]	[g_N_·m^−2^·d^−1^]	
**Partial nitrification**						
PBBR	6	27	700	< 50	< 0.7	Feng et al. (2008)
MABR 1	6.2-7	23	2390	268	1.8	Udert and Wächter (2012)
MBBR	5.8	26	1790	640	2.1	Fumasoli et al. (2016)
MBBR	6.0	23	4140	120	0.4	Fumasoli et al. (2016)
**Complete nitrification**						
PBBR	8	27	600	< 50	< 0.7	Feng et al. (2008)
Biofilm 2	7	15-25	800	< 50	< 1.8	Oosterhuis and van Loosdrecht (2009)
SBR 3	7.3-7.6	25	1500	1100	-4	Jiang et al. (2011)
SBR 3	≥ 6.5	-	1270^5^	600-700	0.6-0.7	Mackey et al. (2014)
MBR	6.9-7.1	-	6300	225^6^	-	Coppens et al. (2016)
MBBR	6.7-6.8	22-23	1080-2160	197^6^	-7	De Paepe et al. (2018)

1 Hybrid, with bubble aeration

2 Vertical sheets

3 Granules

4 No biofilm process

5 Urine diluted four times with seawater

6 Loading rate not optimized

7 No surface rate calculated because both biofilm and suspended biomass contributed to the degradation

#### 3.1.2 Complete nitrification of urine: Biological production of nitrate

Complete nitrification, i.e. oxidation of all ammonia to nitrate, is only possible by providing additional alkalinity. This has been proven in biofilm systems as well as in reactors with suspended biomass, at a pH between 6.5 and 8 (Table 2). Like in the case of partial nitrification (section 3.1.1), we observe higher nitrification rates at higher dilution of urine. Coppens et al. (2016) showed the importance of a halotolerant inoculum, which halved the start-up time as compared to an inoculum from a WWTP. Unfortunately, this did not lead to a higher final reaction rate, indicating that long-term adaptation to high salt concentrations is difficult, especially for the ammonia oxidizers, identified by the authors as less tolerant to high salt concentrations than the nitrite oxidizers. The observed inhibition by salt is supported by the comparable low process rates found by Mackey et al. (2014), who diluted urine with seawater instead of tap water as done in comparable experiments (Jiang et al. 2011). For many practical purposes of complete nitrification, especially heterotrophic denitrification in sewers, dilution with tap water is unproblematic and the optimal dilution is therefore of less importance than for partial nitrification, where volume reduction is most often intended (see section 3.1.1 for a discussion of the trade-offs between dilution and volume reduction). In fact, using a larger water volume for urine flushing will easily achieve sufficient dilution of ammonia and salt, except in places like Hong Kong, where toilets are flushed with seawater. For practical implementation, especially in on-site settings, dosing of alkalinity is the main challenge (Oosterhuis and van Loosdrecht 2009).

### 3.2 Chemical processes for stabilization

Chemical stabilization of urea inhibits the enzyme responsible for its degradation and prevents a pH-increase in the first place (equation 1). Ray et al. (2018) found that fluoride, ionic zinc and ionic silver were ineffective, whereas acid proved effective as already discussed by Maurer et al. (2006). The technologies of alkaline (section 3.2.2) and electrochemical stabilization (section 3.3) have only emerged after 2006. Little new literature is available on neutralization of stored urine with acid (section 3.2.1) and filtration and precipitation to prevent fouling of synthetic membranes (included in section 4.2).

#### 3.2.1 Acid dosage

While Antonini et al. (2012) found neutralization of stored urine with strong acid effective, but too dangerous to implement in a simple setting in Vietnam, Jiang et al. (2017) established a quantitative relationship between pH-value and ammonia loss for a distillation process on acidified stored urine, with 99.5% N-recovery at pH < 4, and less than 50% at pH > 7. As already discussed in Maurer et al. (2006), however, neutralization of stored urine requires large amounts of acid due to the high alkalinity of urine after urea hydrolysis (Table 1). For the more economic urea stabilization in fresh urine, Ray et al. (2018) found that weak acids like acetic acid, citric acid, and vinegar could be used for the inhibition of urea hydrolysis in the concentration range of 32-130 meq·L^−1^. For short-term inhibition of urea hydrolysis in a building urine-collecting system, Saetta et al. (2019) used predictive modeling combined with real-time conductivity and pH data for dosing acetic acid in order to keep a low pH around 4. Low urination volumes and frequencies led to the highest degree of hydrolysis, i.e. the highest demand for acetic acid, whereas high urination volumes and frequency led to less mixing and less time for hydrolysis in the pipes.

In the International Space Station (ISS), sulfuric acid has been used for stabilization, in combination with 0.56% chromium trioxide, but even these harsh conditions could not totally prevent fungal growth, which was the primary cause for biofouling and damaged hardware observed in the urine processing assembly (Birmele et al. 2009a, Birmele et al. 2009b).

#### 3.2.2 Base dosage

Alkaline stabilization of urea is the alternative to acidic stabilization. Randall et al. (2016) compared three chemicals for reaching a pH above 11, necessary for urease inhibition in urine: calcium oxide, calcium carbonate, and slaked lime. Of those chemicals, slaked lime, i.e. Ca(OH)_2_, proved to be the only suitable one, with the additional advantage of low cost and low solubility, allowing the right amount to dissolve from a large amount added to a urine container (Randall et al. 2016). An additional advantage of this stabilization process is that it leads to immediate P-precipitation, which may result in the production of a separate P-fertilizer (section 5.2.1). An alternative to direct alkaline stabilization in liquid urine is the same process in a dry bed. Dutta and Vinneras (2016) showed that a mixture of Ca(OH)_2_ and wood ash (1:1) would keep the pH value of fresh urine above 10, which in this solid mixture was deemed enough to retain urea, although some ammonia losses occurred. Senecal and Vinneras (2017) even reached pH values higher than 10 with wood ash alone. Simha et al. (2018a) showed that the concept of an anion-exchanger resin could potentially work, exchanging OH^−^ ions against Cl^−^ ions for providing additional base to fresh urine prior to these processes, but up to 2019, we have seen no follow-up projects.

An aspect of high significance for liquid and dry alkaline stabilization alike is the stability of the pH value once obtained. Senecal and Vinneras (2017) observed that CO_2_ adsorption from the air led to a critical pH decrease. Simha et al. (2018a) suggested that besides CO_2_ absorption, buffering of alkaline earth metals and NH_tot_ formation from urea degradation lead to a pH decrease. The most important research gap for alkaline stabilization is thus the setup of a system, which effectively prevents CO_2_ uptake from air, thereby inducing a critical positive feedback mechanism due to increased urea hydrolysis.

In section 4, we will discuss the volume reduction of stabilized urine for fertilizer production. An alternative usage for alkaline stabilized urine would be the production of bricks through mixing with sand and urease-producing base-tolerant bacteria (Henze and Randall 2018, Lambert and Randall 2019). The carbonate produced through urea hydrolysis combines with calcium and the resulting calcium carbonate cements the sand particles together, allowing for the production of a low-cost building material with a compressive strength comparable to the strength of conventional bricks.

### 3.3 Electrochemical processes for stabilization

Electrochemical processes have been used to stabilize urine by removing organic substances, preventing urea hydrolysis or inactivating microorganisms. In this section, we discuss mainly urine stabilization in electrolysis cells (EC). Removal of organic substances has also been investigated to a large extent with microbial fuel cells (MFC) with the benefit of producing electricity (see section 7).

In most studies on urine electrolysis, indirect oxidation with chlorine is the main mechanism for removal of organics and nitrogen compounds, and sometimes, NaCl was even added to boost the oxidation process (Chun et al. 2018, Ikematsu et al. 2006). Chlorine is a potent oxidant, which is produced by the oxidation of chloride. Besides chlorine, hydroxyl radicals are important oxidants, especially in the case of boron-doped diamond electrodes (BDD).

Zöllig et al. (2017) conducted batch experiments in the lab to investigate the electrolysis of real stored urine using either BDD or a titanium anode with a thermally decomposed IrO_2_ film (TDIROF) (see also section 6.2 on nitrogen removal). On both electrodes, fast removal of organic substances started right from the beginning of the experiments. Actually, at the start of the experiment COD removal was preferred over ammonia oxidation on BDD. The COD degradation rates were 10 to 50 times higher than rates observed in biofilm systems or in MFC (see Table 3). However, the energy demand is very high. Actually, MFC might be an interesting alternative for COD removal. The degradation rates are similar to the maximum rates observed in conventional biofilm systems, and the electricity produced could be used for process monitoring.

**Table 3:** Typical values for COD-removal in electrochemical systems, compared to conventional biofilm systems

Process	Degradation rate	Energy demand for oxidation	CBP	Reference
	[g_COD_·m^−2^·d^−1^]	[kWh·kg_COD_^−1^]	[-]	
Electrolysis on BDD 1^,^^2^	421	55	Yes	Zöllig et al. (2017)
Electrolysis on TDIROF 1^,^^3^	214	67	Yes	Zöllig et al. (2017)
MFC 4	41	-0.56	No	
Conventional biofilm system 5	8-40	depends on aeration	No	Sørensen and Morgenroth (2020)

1 20 mA·m^−2^, Batch experiments

2 90% COD removal

3 30% COD removal

4 See Table 11 for calculation

5 Assuming 2 g_COD_·g_BOD_^−1^ (Metcalf&Eddy 2014)

While indirect oxidation with free chlorine can be a fast and efficient removal process for organics, nitrogen compounds (section 6.2), pharmaceuticals and pathogens (section 8), the process can be problematic due to the formation of chlorinated by-products (CBPs), which can be hazardous for the environment and human health (Zöllig et al. 2015c). Dbira et al. (2015) and Zöllig et al. (2017) reported that chloride was nearly completely removed, due to the formation of CBPs. Zöllig et al. (2015c) examined the fate of the organic CBPs dichloromethane, trichloromethane, tetrachloromethane, 1,2-dichloroethane, 1,2-dichloropropane and chlorobenzene and the inorganic CBPs chlorate and perchlorate during electrolysis on BDD and TDIROF. Perchlorate and chlorate were the dominant CBPs (consuming more than 90% of the initial chloride). Most of the organic CBPs were stripped to the air thereby posing a health hazard.

## 4 Nutrient recovery by volume reduction

Volume reduction typically results in the recovery of several nutrients. The technologies of interest rely on evaporation, membrane processes including ED (a combination of membrane technology and electrochemistry), and the uptake of nutrients in algae.

### 4.1 Drying and distillation processes

Distillation and drying both rely on evaporation, but whereas water in the distillation process is re-condensed – with the possibility to recover water and some of the high evaporation energy of around 700 Wh·L^−1^ (Udert and Wächter 2012), this normally does not happen in a drying process. Evaporation essentially concentrates all non-volatile compounds in urine, with the large advantage of keeping also micronutrients in the fertilizer product (Harder et al. 2019), but also with potential negative consequences if organic micropollutants are not removed along the process chain (Section 8.2). There is always a risk of some ammonia volatilization, but depending on the effectivity of the preceding stabilization process (section 3) and the process configuration, this loss can be minimized.

Drying has mainly been suggested for on-site settings in the bathroom after on-site alkaline stabilization. The process can profit from high air temperatures, but as shown by Randall et al. (2016), alkaline stabilized urea becomes chemically unstable at temperatures above 40°C. Dutta and Vinneras (2016) and Senecal and Vinneras (2017) evaporated alkaline stabilized urine by applying a forced air stream to a drying bed based on wood ash. Senecal and Vinneras (2017) obtained more than 80% nitrogen recovery at an evaporation temperature of 35°C, resulting in a solid product with up to 7.8% N, 2.5% P and 10.9% K by weight, comparable to commercial NPK fertilizers. The corresponding high loss of ammonia to the atmosphere obviously calls for process improvements. In a similar study, Simha et al. (2018a) established that higher temperatures led to higher evaporation rates, but that a higher air flow rate was only beneficial within limits and a deeper bed was counterproductive. Due to the instability of urea at temperatures above 40°C, there are clear limits for the improvement of rates based on temperature.

Udert and Wächter (2012) investigated water removal from partially nitrified urine (section 3.1.1) by distillation in a small lab-scale evaporator at 78°C and 200 mbar, resulting in a dry product or a highly concentrated liquid product. Fumasoli et al. (2016) describe the application of this process in a commercial distillation reactor with vapor compression and heat recovery, resulting in a concentrated nutrient solution with concentrations (w/w) of around 5% N, 0.2% P and 2% K. The energy demand of distillation was around 110 Wh·L^−1^, as compared to around 710 Wh·L^−1^ for the evaporation of water without energy recovery. Equally important is that the small loss of nitrogen is contained in the distillate and therefore not emitted to the atmosphere. Distillation of acid-stabilized fresh urine is possible (Carter et al. 2013), as is distillation of fresh urine without stabilization (Lefebvre et al. 2015), but in the latter case obviously only for a short time until urea hydrolysis becomes substantial. We have found no attempts to use distillation for urine stabilized at a high pH-value, though high ammonia losses are to be expected due to chemical urea hydrolysis at high temperatures and high pH values (Randall et al. 2016).

The advantage of drying is the applicability at bathroom scale, but there are still important research gaps with respect to energy recovery and reduction of ammonia losses. The obvious advantages of distillation is energy recovery and the capture of ammonia in the distillate, where it will not lead to air contamination. Distillation is a well-established commercial process, but if it is to be downscaled, more explorative processes like membrane distillation (MD; section 4.2.2) could be more promising.

### 4.2 Membrane processes

The membrane processes applied for volume reduction are primarily forward osmosis (FO) and ED. For the latter, we refer to section 4.3. Further, MD is an explorative alternative technology to conventional distillation (section 4.1). Ultrafiltration has proven an effective pre-treatment step prior to the osmotic membrane processes intended for volume reduction, removing up to 99% of the suspended solids in stored urine, but with severe fouling problems of the membrane (Ouma et al. 2016).

For all membrane processes, there are important differences between the treatment of fresh urine with nitrogen mainly contained in urea, an uncharged molecule, and the treatment of stored urine, where nitrogen is mainly contained in the acid-base pair NH_4_^+^/NH_3_. All membrane applications suffer from fouling, leading in many cases to short duration of the experiments. This will be a major hurdle for on-site applications of membrane treatment, where regular cleaning of the membranes is difficult.

#### 4.2.1 Forward Osmosis (FO)

With the technology of FO, the rejection of nitrogen compounds (Volpin et al. 2019) *and* the water flux (Liu et al. 2016) increase with increasing concentration of the draw solution. From Table 4, summarizing the available results on the process, we observe that high retention of all three main nutrients (N, P and K) is possible as well as a high volume reduction of up to 85%. However, all authors applied the process only to synthetic urine and none obtained good results on all parameters. Those who used fresh urine, report pH values of 6-7, where urea is not stable (section 3.2). For stored urine, we only observe good ammonia retention at a low pH-values, at which the concentration of NH_3_ is low, requiring large amounts of acid for neutralization (Maurer et al. 2006). All authors identified fouling as a major problem. Zhang et al. (2014) reduced the problem by removal of precipitates prior to membrane treatment, but only for an experimental duration of 70 hours.

**Table 4:** Forward osmosis (FO) processes for volume reduction of urine, at temperatures of 18-25°C, using a NaCl draw solution, with a duration T_d_ of the experiments, all conducted with synthetic urine. Volume reduction, ΔV, is only relevant for a few experiments set up to deliver relevant results on this parameter.

C_N,tot,in_	T_d_	Draw solution	pH	Flow rate	ΔV	Retention [%]				Reference
[g_N_·L^−1^]	[h]	[mol·L-^1^]	[-]	[L·m^−2^·h^−1^]	[%]	Urea	NH_tot_	P	K	
Fresh urine										
2.4-12	70	2	7	max. 20	60-85	<50	-	97-99	79-97	Zhang et al. (2014)
7.5	2	1-2.5	6	3-6	-	98	-	-	-	Liu et al. (2016)
4.2	2	2.5	6.2	31.5	-	80	-	-	-	Volpin et al. (2019)
Stored urine										
2.4-12	70	2	9.3	max. 20	60-85	-	40-60	97-99	79-97	Zhang et al. (2014)
5.6	2	2.5	6	28.7	-	-	>95	-	-	Volpin et al. (2019)

#### 4.2.2 Membrane Distillation (MD)

In the MD process, the driving force is the vapor pressure difference produced by the temperature difference across a hydrophobic membrane, only allowing volatile compounds to pass (Derese and Verliefde 2016). From urine, we would thus expect water vapor, NH_3_, and volatile organic compounds to pass the membrane, making the process suitable only for stabilized urine, where the concentration of free ammonia is low. As we will see, however, some new membrane developments may challenge this conventional wisdom. The energy demand for MD is slightly higher than for vapor compression distillation with heat recovery. For example, Winter et al. (2011) reported energy demands of 180 to 240 kWh·m^−3^ for treating water with a salinity of 35 g·kg^−1^ in spiral wound MD modules, while Fumasoli et al. (2016) reported 107 kWh·m^−3^ for treating partially nitrified urine with vapor compression distillation and heat recovery. The advantage of MD over conventional vapor-compression distillation could be the simpler setup and the possibility to use low-grade heat instead of electricity (Derese and Verliefde 2016).

There is little experience with MD of stabilized fresh urine. Ray et al. (2019) came close by testing DCMD on a urea solution previously obtained from urine through an FO process. The FO process resulted in (incomplete) selective mass transfer of urea, but with some co-transfer of COD. In the MD process on the urea solution, urea recovery ranged from 72-92%, and different stabilization methods had no effects on recovery. Despite the reduced COD-content, severe fouling of the membrane occurred, strongly questioning the viability of MD on fresh urine. For stored urine, Tun et al. (2016) showed that the permeation of nitrogen through the membrane is proportional to the concentration of free ammonia in the feed solution and negligible at pH ≤ 6 following chemical acidification. The authors applied filtration to prevent fouling, but with little effect. Xu et al. (2019a) suggested partial or full nitrification for stabilization, which would have the additional advantage of removing a large portion of the COD. With an unusual high pH of 8.3 after partial nitrification (see section 3.1.1), only complete nitrification (section 3.1.2) resulted in a high nitrogen retention of 94%, but the experiments were too short to verify the anti-fouling effects of biological treatment.

In general, a major research gap for FO is the lack of long-term experience, especially with respect to fouling. Additionally, like for the FO processes, only stored urine with a low pH is suitable as feed, requiring large amounts of acid for neutralization (Maurer et al. 2006). In an alternative approach to deal with the poor rejection of ammonia by DCMD at high pH, Khumalo et al. (2019) changed the membrane properties with nanoparticles, creating smaller evenly distributed pores and a porous spongy structure. In experiments on real stored urine without pH adjustment, with a temperature difference of 30°C, and using the most hydrophobic membranes produced, the authors found 80% water recovery, with a rejection rate for ammonia higher than 95% and similar rejection rates for total organic carbon (TOC), potassium and sodium ions. The authors attributed these highly interesting results to the dense porous structure, which is able to trap the molecules during their travel through the membrane. With reported low fouling-potential and stable water flux rates over a period of 15 days, further research on these modified membranes seems worthwhile.

### 4.3 Electrodialysis for volume reduction

ED has been used to produce a concentrated solution of all ions in urine. An electric field and at least one pair of an anion exchange membrane (AEM) and a cation-exchange membrane (CEM) is needed in ED to concentrate all ions, while for ammonium removal via ED only a CEM is needed (see section 5.1.3).

In most studies on urine ED, urine was in direct contact with the anode and the cathode. The required voltage was produced by oxidizing reduced compounds, especially COD. The reactors were operated as microbial fuel cells (MFC-ED; Freguia et al. (2019), Gao et al. (2018b), Lu et al. (2019)), microbial electrolysis cells (MEC-ED; Ledezma et al. (2017), Brewster et al. (2017), Tice and Kim (2014)) or electrolysis cells (EC-ED; Jermakka et al. (2018)). In EC, high voltages can result in unwanted CBPs (see section 3.3). Pronk et al. (2007) and De Paepe et al. (2018) used conventional electrodialysis, and rinsed the electrodes with a sodium sulfate or sodium nitrate solution, respectively.

In all ED processes, except for MFC, electric energy is needed to apply an external voltage. In general, the energy demand was low as compared to distillation (see Table 5). The lowest energy demand observed was 1.3 kWh·kg_N-recovered_^−1^ when concentrating diluted nitrified urine with conventional ED (calculated from data by De Paepe et al. (2018)).

**Table 5:** Typical values for electrodialysis (ED) performance, compared to distillation

Process	Feed	Concentration factor			Nutrient loss			Energy demand	Reference
		N 1	P	K	N 1	P	K		
		[-]	[-]	[-]	[%]	[%]	[%]	[kWh·kg_N-recovered_^−1^]	
ED 2	6	4.3	2.6	4.6	30	60	29	1.3	De Paepe et al. (2018)
MEC-ED 3	7	4.5	12.2	3.8	50	57	45	2.4	Ledezma et al. (2017)
EC-ED 4	7	4.1	3.2	4.5	28	61	21	13	Jermakka et al. (2018)
Distillation 5	8	11	9	12	0.6	0	0	26	Fumasoli et al. (2016), Udert and Wächter (2012)

1 NH_tot_ or nitrate

2 Conventional ED, 0.05 A or 7,800 mA·m^−2^ assuming a surface area of 64 cm^2^, 10 cell pairs

3 29 mA·m^−2^, 1 cell pair

4 100 mA·m^−2^, 1 cell pair

5 107 kWh·m^−3^, 4.1 g_N_·m^−3^ NH_4_NO_3_

6 Completely nitrified real urine, 5 times diluted

7 Synthetic stored urine, undiluted

8 Partially nitrified urine, undiluted

Despite good concentration factors (see Table 5), up to 50% of nitrogen, 61% of the phosphate and 45% of the potassium was lost, mainly to the diluate. Higher concentrations factors are not to be expected, because volume reduction is limited by osmotic and electro-osmotic water transport into the concentrate (Pronk et al. 2006a) and back diffusion of ions from the concentrate into the diluate (Brewster et al., 2017). Besides lower energy demand, removal of organic micropollutants is an advantage of ED compared to distillation (see section 8.2.3). However, it comes at the cost of a large share of the nutrients remaining in the diluate. Furthermore, as for all membrane processes, fouling is a problem. To prevent fouling, Pronk et al. (2007) and De Paepe et al. (2018) pre-treated the influent with microfiltration. In addition, De Paepe et al. (2018) cleaned the membranes once a month.

### 4.4 Volume reduction through nutrient uptake in algae

A biological process for volume reduction would be the uptake of nutrients in algae, followed by separation and possibly a drying process. With typical nitrogen concentrations (w/w) of 0.92 % in urine (Table 1) and 6-8 % in algae dry matter (Tuantet et al. 2013), and with typical algae dry matter contents around 25 % (Da Silva et al. 2008), this would result in weight reductions of only a factor of 2 for wet, but a factor of 8 for dried algae. However, in the literature, we have found only experimental results for algae growth on urine, which is already quite complex, but no discussion of the following separation and drying processes.

In several studies, urine functions as a nutrient source for producing algae, either for use as a slow-release fertilizer, as an energy crop, or for the production of other chemical products from algae. Despite a large variability in the literature with respect to urine dilution, type of algae species and reactor operation, the requirement for a culture medium additional to urine is universal. Urine cannot on its own support substantial algae growth due to a lack of micronutrients and a high N:P ratio in fresh urine of approximately 28:1 as compared to the general requirements of algae of 16:1, leading to the requirement of P-addition for quantitative N-removal (Tuantet 2015). Precipitation of P during storage further increases the N:P ratio and removes essential compounds like Mg and Ca (section 2).

Like biological stabilization (section 3.1), algae growth suffers from inhibition through salt and nitrogen compounds, especially free ammonia above 140 g_N_·m^−3^ (Tuantet et al. 2013), but also high nitrate concentrations above 1000 g_N_·m^−3^ (Coppens et al. 2016). Undiluted urine is therefore not a suitable substrate. The best long-term results were obtained by Tuantet et al. (2014), who optimized a continuous photo bioreactor for more than 8 months at a pH of 7, with a minimum dilution factor of 2 and the addition of the missing nutrients. The maximum N-uptake rate was 1300 g_N_·m^−3^·d^−1^ and the maximum P-uptake rate 150 g_P_·m^−3^·d^−1^.

In addition to the rather unsuitable nutrient composition of urine for algae growth, the setup of bioreactors is challenging due to low light penetration, aggravated by the dark color of urine (Coppens et al. 2016). To overcome inhibition and improve light penetration, bioreactors are typically set up for diluted urine. Tuantet et al. (2019) showed that continuous microalgae cultivation in a photobioreactor with a light path of 5 mm is possible, but that there is an inherent conflict between quantitative nitrogen removal and photosynthetic efficiency when treating urine at reasonable nitrogen concentrations between 0.77 and 2.6 g·L^−1^. The shorter the hydraulic retention time (HRT), the higher the dilution factor of urine in order to prevent inhibition of the algae by free ammonia. With increasing HRT, both biomass concentration and nutrient removal increases, but at a certain point depending on the length of the light path, photosynthetic efficiency decreases due to the high biomass concentration. Optimizing the system would thus demand even shorter light paths than used in this study, an enormous challenge for the open raceway ponds normally suggested for algae growth. This was confirmed by Chatterjee et al. (2019) in a pilot study of a 0.5 m deep raceway pond, where extremely high dilution was required even for only 50% nitrogen recovery. Based on the daily nitrogen excretion in urine reported in section 2, such a plant would have a footprint of 3 m^2^·p^−1^, i.e. 15 ha for a middle-sized town of 50,000 inhabitants, and is therefore hardly a realistic option. Apart from the challenges concerning post-processing, the development of a realistic photobioreactor is thus the most important research gap in the area of volume reduction of urine through algae growth.

## 5 Targeted Nutrient Recovery

Targeted nutrient recovery is primarily directed at the pollution-relevant nutrients nitrogen (N) and phosphorus (P), while only little efforts have been directed towards potassium (K).

### 5.1 Targeted N-recovery

Nitrogen is the most important nutrient in urine: it has the highest economic value (Etter et al. 2011) and it is responsible for the increased size and complexity of nutrient-eliminating plants, as well as for most of the climate effects (energy demand and N_2_O emissions; Gruber et al. (2020), Larsen (2015)). Based on four papers on urine treatment, Maurer et al. (2006) presented three technologies for targeted nitrogen recovery: Air stripping followed by ammonia absorption in distilled water, ion-exchange on zeolites and precipitation with Isobutylaldehyde-diurea (IBDU). While the IBDU precipitation was inefficient and did not lead to any follow-up projects, the technologies of air stripping and ion exchange have developed considerably since then.

#### 5.1.1 Stripping for targeted N-recovery

Stored urine with its high concentration of ammonia at a pH around 9.3 (section 2) is highly favorable for an air-stripping process. Morales et al. (2013) showed that stored urine can be added to digester supernatant for ammonia removal, but the high phosphate concentrations in urine can lead to critical precipitation. Table 6 provides an overview of literature results on air stripping of nitrogen from urine. P-removal usually precedes the stripping process to prevent problems by clogging.

**Table 6:** Direct air-stripping of ammonia from real urine, with absorption in H_2_SO_4_. Results are reported for optimal conditions, after P-removal. Recovery is given in percent of removed nitrogen.

Reactor set-up	pH	T	Removal	H_2_SO_4_	Recovery	Energy demand	Reference
	[-]	[°C]	[%]	[mol·L^−1^]	[%]	[kWh·kg_N-recovered_^−1^]	
Batch	12	16	93	0.5	92	-	Basakçılardan-Kabakcı et al. (2007)
Continuous 1^,^^2^	10	40	94	1.9	∼ 100	19-28	Antonini et al. (2011)
Batch 3	9.3	50	80	1	90-95	29	Liu et al. (2015)
Batch	> 12	30-40	85-99	1^4^	94-99	15-20	Pradhan et al. (2017)
Continuous 1	10.6-11	35	> 99	2	79	-	Xu et al. (2017)
Continuous 1^,^^5^	9	35-45	92-93	18	93	7	Wei et al. (2018)
Continuous 1^,^^6^	9.2	22	87	3	31	6	Christiaens et al. (2017)

1 Stripping and absorber column

2 With air and urine recirculation

3 Air-stripping tower run in batch mode

4 In two serial tanks and augmented with 9 M H_2_SO_4_ to keep pH < 2

5 With air recirculation

6 Increase of pH in cathode chamber of an ED module

Air stripping of ammonia is facilitated by high pH and high temperature, both pushing the acid-base equilibrium towards volatile free ammonia (Basakçılardan-Kabakcı et al. 2007, Liu et al. 2015). As observed from Table 6, different authors have different priorities with respect to resource demand, i.e. chemicals for pH-increase and energy, and removal efficiency of the process. In some cases, there are special reasons for these priorities. Pradhan et al. (2017), for instance, combined P-removal and N-stripping by increasing pH with Ca(OH)_2_, thereby inducing the precipitation of calcium phosphates (section 5.2.1). In a combination of experimental work and modeling, Liu et al. (2015) found a strong positive correlation of air stripping efficiency within the following boundaries: pH 9.3-10, temperature 23-50°C, and air flow-rate 1-4 L_air_·h^−1^·L_urine_^−1^. Dilution hardly influenced the percentage of ammonia removal, indicating an approximate linear correlation between ammonia concentration and process efficiency, assuming that resource consumption is more or less proportional to the volume stream treated. The model provides a first useful tool to weigh resource demand against nitrogen stripping efficiency according to the preferences of the stakeholders.

Emerging technologies challenging conventional ammonia stripping are vacuum thermal stripping (Tao et al. 2019, Tian et al. 2019), membrane stripping (Christiaens et al. 2019b, Pradhan et al. 2019), and MD (Derese and Verliefde 2016). Common for these technologies is that they do not need an air stream for ammonia transfer, potentially simplifying the technical setup, but all technologies are still experimental. Christiaens et al. (2019b) performed a comparison with conventional stripping and found that direct liquid-liquid transfer from a concentrated urine solution through a hydrophobic gas membrane could halve the energy demand required for the same result from air stripping.

For conventional and emerging stripping technologies, the typical absorption medium is sulfuric acid. Boehler et al. (2015) recommend a 14 M H_2_SO_4_ solution to reach a 40% salt solution with 100 g_N_·L^−1^, avoiding crystallization of ammonium sulfate. With around 9.2 g_N_·L^−1^ in undiluted urine (Table 1), this corresponds to a volume reduction of more than a factor of 10. However, many authors use 0.5-2 M acids, leading to highly dilute solutions (Table 6), and a higher stripping temperature leads to a higher co-transfer rate of water (Pradhan et al. 2017). In the case of vacuum thermal stripping, co-evaporation is so high that Tao et al. (2019) successfully installed a demister, resulting in crystallization even in a 2.5 M H_2_SO_4_ solution. Efficient ammonia recovery requires low pH values and typically, the pH value of the absorber medium is used for control. However, for instance Xu et al. (2017) only stopped the adsorption process at pH 9.3, which resulted in 24% nitrogen loss to the atmosphere.

For air-stripping of ammonia, a combination of counter-currently operated packed column stripper and absorber is standard technology. Antonini et al. (2011) successfully improved the design by introducing recirculation of the air stream between the two columns to avoid nitrogen losses, and recirculation of urine around the stripping column to improve mass transfer. Pradhan et al. (2017) reduced ammonia loss by installing two serial absorbers. We only found one example of a pilot plant, running successful for 80 days (Wei et al. 2018), achieving more than 90 % NH_tot_ removal at an HRT in the order of 5.5-6.3 minutes independently of dilution. However, despite air-recirculation as recommended above, nitrogen recovery was only 93%.

The main critical issue of stripping, however, is the required large scale of application. Although Antonini et al. (2011) applied a flow rate as low as 10 L·h^−1^ of undiluted urine, this flow rate applied 24/7 would correspond to treating the urine from more than 150 people. The only long-term pilot plant was run at a loading rate of 7-8 m^3^·h^−1^ of urine diluted by a factor of 4 (Wei et al. 2018), which would correspond to treating the urine of more than 30 000 people. In order to be relevant at the scale of an apartment building, a small-scale column setup and automatic control system would be necessary. While the emerging alternatives discussed above avoid at least the air-stripping column and therefore seem simpler and better suited for small-scale application, these technologies are still only experimental.

#### 5.1.2 Adsorption processes for targeted N-recovery

Whereas ammonia stripping is associated with large-scale columns, the adsorption technology with no moving parts seems perfect for small-scale applications. Maurer et al. (2006) already reported first experience with ion-exchange on zeolites for targeted NH_tot_ recovery from stored urine, and in fact, zeolites are still by far the preferred adsorption material for targeted N-recovery. Because zeolites are excellent soil conditioners for increasing nutrient and water retention of poor soils (Ramesh and Reddy 2011), the product is always intended directly as fertilizer, as is biochar, another absorber material used for nitrogen recovery. For a thorough discussion of adsorption mechanisms and an overview of the experimental results, see the excellent review of Kabdaşlı and Tünay (2018).

Only Tarpeh et al. (2017) have tested other adsorbents for nitrogen recovery (Table 7). For biochar, maximum adsorption densities in real urine is similar to the ones for zeolites, whereas some synthetic cation exchange resins were more effective, reaching a maximal ammonium adsorption density of up to 64 mg_N_·g^−1^, a factor 7 weight reduction. This is substantial and although slightly lower than for stripping, the technology is simpler for small-scale installations. The price for the higher adsorption capacity is the requirement for a subsequent regeneration step, preferably with a strong acid. With the extent of desorption widely depending on the adsorbent material (Tarpeh et al. 2017), this also sets a limit to the lifetime of the synthetic material. There are few examples of an installation of ion exchangers beyond the lab. Recently, Tarpeh et al. (2018b) did a first successful pilot installation of the synthetic resin Dowex Mac 3 for ammonium recovery in Kenya, in cooperation with the social enterprise Sanergy. This first pilot phase showed similar adsorption densities as in the lab, but only a longer-term installation will reveal the sustainability of the business model.

**Table 7:** Average maximum ammonium adsorption densities from real stored urine on different resins (Tarpeh et al. 2017).

Adsorbent	Max adsorption density	
	[g_N_·kg^−1^]	[mol_N_·kg^−1^]
Natural zeolites	32	2.3
Biochar	29	2.1
Dowex 50	45	3.2
Dowex MAC 3	56	4.0

Nitrogen in the form of urea is the only non-ionic nutrient in urine. Until now, one group of researchers have tested the adsorption of urea to biochar, with results ranging from highly surprising 750 mg_urea_·g^−1^ in batch experiments (Ganesapillai et al. 2015) to more realistic 94 mg_urea_·g^−1^ (44 mg_N_·g^−1^) in column experiments (Simha et al. 2018b). These and similar experiments were all conducted at neutral pH, where urea is not stable, and some hydrolysis may have interfered with the urea measurements. For practical purposes, results from urea-stabilized urine (section 3.2) will be required in order to compare adsorption of urea and ammonium on biochar (the latter as reported in Table 7) and to evaluate whether the additional stabilization is worth the effort.

#### 5.1.3 Electrochemical processes for targeted N-recovery

**Fig. 1:**
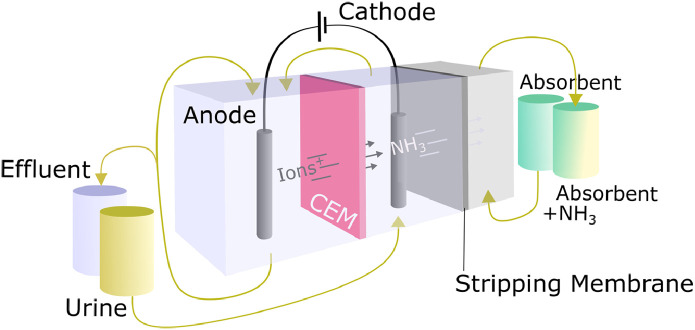
Example of a combined electrochemical cell for ammonia concentration and ammonia stripping. Alternatives A1: stripping column and A2: membrane stripping. Based on Christiaens et al. (2019b).

ED has frequently been used to move ammonium from urine towards a cathode for subsequent recovery. In this application of ED, only a CEM is needed, which allows the selective migration of cations including ammonium, to the cathode (Fig. 1). The high pH value at the cathode can then be used for ammonia stripping either with air or through membranes (see section 5.1.1). The electric field required for ED can be produced with a MFC (Kuntke et al. 2012), MEC (Kuntke et al. 2018, Kuntke et al. 2017, Ledezma et al. 2017, Rodríguez Arredondo et al. 2019, Zamora et al. 2017b) or EC system (Christiaens et al. 2019a, Christiaens et al. 2017, Luther et al. 2015, Tarpeh et al. 2018a).

Kuntke et al. (2012) reported that the Coulombic efficiency in an MFC was only 10%, i.e. only 10% of the electrons released from COD degradation contributed to the electric current and thereby to cation transport in the electric field. Furthermore, only 31% of the current was used for ammonium transport, while most of the current was used to transport other cations, such as sodium, potassium and protons (Kuntke et al. 2017). However, the low transport of ammonium was somewhat compensated by the diffusion of uncharged free ammonia. In the study of Kuntke et al. (2012), at least 42% of the NH_tot_ was transported via free ammonia diffusion. The contribution of free ammonia diffusion became actually the dominant ammonia transport mechanism at low current densities.

The same research group showed that the electro-migration of ammonium can be substantially enhanced with MECs. Zamora et al. (2017a) reported that 92% of NH_tot_ was transported through the CEM. This high electro-migration of ammonium correlated with a high Coulombic efficiency of 70%. The process was also faster than in the MFC study (Kuntke et al. 2012) because the current densities were up to 100 times higher (50 A·m^−2^).

After moving the ammonium to the cathode, it must be stripped from the cathode chamber by air stripping (e.g. Kuntke et al. (2012)) or through a membrane to be absorbed in an acid (e.g. Zamora et al. (2017a) and Tarpeh et al. (2018a)). The study by Zamora et al. (2017a) showed that the limiting step for final ammonia recovery is not the removal of ammonia from urine but the capture of the removed ammonium in the acid. While 92% of the total ammonia was transported through the CEM, only 31% was finally absorbed in sulfuric acid. The highest NH_tot_ recovery for real urine was reported by Tarpeh et al. (2018a). When combining an EC-ED with ammonia stripping through gas permeable membranes, they could recover 93% of the NH_tot_ in the acid in batch mode and 50% in continuous mode. However, they also showed that substantial amounts of ammonia can be lost by the oxidation with chlorine, which can be formed at the anode. Kuntke et al. (2017) actually presented a method to prevent chlorine formation at the anode. By recycling hydrogen from the cathode to the anode, high anode potentials could be prevented and thereby the production of chlorine and CBPs. Furthermore, the availability of hydrogen also reduced the consumption of organic compounds at the anode.

In most studies on electrochemical ammonia stripping, a synthetic catholyte was used. Only in two studies (Christiaens et al. 2017, Christiaens et al. 2019b), was urine treated directly at the cathode. After entering the cathode chamber, it was directed into the anode chamber. Ammonia was later absorbed in sulfuric acid or in a solution with hydrogen oxidizing bacteria to produce microbial protein (Christiaens et al. 2017).

### 5.2 Targeted P-recovery

Based on the experience from conventional wastewater treatment, precipitation is the natural process choice for targeted P-recovery from urine. Although also anion exchange has been attempted, it is not always clear if in fact ion exchange and not precipitation is at least partially responsible for the removal effects.

#### 5.2.1 Precipitation processes for targeted P-recovery

In wastewater, phosphate precipitates with iron, aluminum, magnesium, and calcium ions. These ions have equally proven successful for recovering phosphorus from urine, but precipitation with Mg^2+^ is by far the most popular process for this purpose. One reason for this may be the large focus on P-recovery for fertilizer. Whereas Magnesium Ammonium Phosphate (MAP) is recognized as a good plant fertilizer (Harder et al. 2019), plant availability of iron and aluminum phosphate and possible toxicity of the latter is heavily debated (see e.g. Mocker et al. (2011)). However, these simple technologies may prove suitable for collecting P at the source for later industrial fertilizer production. Table 8 lists the best results obtained from the literature for precipitation processes for P-recovery from urine.

**Table 8:** Best results reported on chemical precipitation processes for phosphorus recovery from urine.

Product	Chemicals 1	pH	Molar ratio	Removal (%)	Reference
MAP	Mg^2+^	8.9-9.3	Mg:P ≈ 1	93-99	Etter et al. (2011), Hug and Udert (2013), Ronteltap et al. (2007b), Schneider et al. (2013), Tao et al. (2019), Wilsenach et al. (2007)
MPP	MgCl_2_	8.2	Mg:P ≈ 2	75	Wilsenach et al. (2007)
MPP	MgO	9.1	Mg:P ≈ 1	95	Wilsenach et al. (2007)
Aluminum phosphate	Al^3^^+^	≤ 6.5	Al:P ≈ 0.7^2^	100	Zheng et al. (2010)
Iron phosphate	Fe^3+^	≤ 9	Fe:P ≈ 1.1	100	Zheng et al. (2009)
Calcium phosphate^3^	Ca^2+^	-	Ca:P high	-	Kabdasli et al. (2019), Pradhan et al. (2019), Pradhan et al. (2017)
Calcium phosphate 4	Ca(OH)_2_	12.4	Ca:P ≈ 2.4	100	Randall et al. (2016)

1 Where the chemical is given only as the relevant ion, different sources have been used or the ion was produced electrochemically (for Al^3+^ and Fe^3+^)

2 Value doubtful.

3 For stored urine

4 For fresh urine, pH increases to 12.4 due to Ca(OH)_2_ dosage. The Ca:P is given for the maximum concentration of P of 0.74 g_P_·L^−1^ and the minimal amount of Ca(OH)_2_ used for stabilization. Ca:P was not optimized for P-precipitation.

For MAP production, the literature is extensive. Experimental evidence shows that particle formation is fast, in the order of less than 20 seconds, supported by the initial presence of small particles (Triger et al. 2012). MAP solubility correlates positively with temperature (Ronteltap et al. 2007b) and particle size increases with decreasing super-saturation (β_S_), i.e. with increasing temperature and decreasing pH, and with increasing turbulence preventing water packets with high super-saturation (Ronteltap et al. 2010). Rodrigues et al. (2019) supported these findings and determined an optimum saturation index (SI = log_10_ (β_S_)) between 2.7 and 3.5 for simultaneously obtaining high phosphorus recovery and large particle sizes, with a higher contact time supporting crystal growth.

For the other precipitation processes, there is substantial less information in the literature. Precipitation of phosphate with aluminum is only successful at low pH values, due to the formation of competing compounds (Table 8). In essence, this means that only stabilized urine is suitable as substrate (section 3). For the production of FePO_4_, there is less agreement in the literature. In experiments using a sacrificial iron electrode for the dosing of iron, Zheng et al. (2009) reported excellent results at pH < 9, i.e. close to the pH of stored urine. In an experiment dosing iron as FeCl_3_, Kabdaşlı et al. (2019) found that above pH 7.5, the formation of Fe(OH)_3_ flocs rendered the process inefficient. In the latter case, again only stabilized urine would be a suitable substrate.

Due to competing CaCO_3_ production, precipitation of phosphate with Ca^2+^ is only recommended for fresh urine, which contains negligible concentrations of bicarbonate (Table 8).

The challenge of a practical reactor setup has been taken up only for MAP and magnesium potassium phosphate (MPP) precipitation (Table 9; see section 5.3 for MPP-precipitation). Scaling problems in CSTRs originally observed by Wilsenach et al. (2007) was solved by initial seeding and the establishment of a more distinct settling zone (Aguado et al. 2019). Particle separation in CSTRs was only a major problem in the study of Wei et al. (2018), where small particles escaped the settling tank as well as the additional sieve. Zamora et al. (2017a) obtained the largest MAP crystals in a four-phased fluidized bed reactor (FBR) reactor, which was specifically designed for crystal growth with a decreasing flow rate along the vertical flow axis, with online measurements of the P-concentration in the influent to determine optimal Mg^2+^-dosing. Zhang et al. (2017) used the same setup for MPP production and obtained even larger crystals at an optimized SI of 3.0. For low-income countries, Etter et al. (2011) developed a low-cost, hand-driven reactor with an external filtration system (coarse nylon filter), which was later automatized with good results by Grau et al. (2015), using online signals of turbidity or electric conductivity for optimizing magnesium dosage.

**Table 9:** Reactor types tested for magnesium ammonium phosphate (MAP) and magnesium potassium phosphate (MPP) precipitation from urine

Reactor type	Number of compartments	Seeding	P-Removal	Crystal size	Reference
CSTR (MAP)	1, but separation zone	no	95%	-	Wilsenach et al. (2007)
CSTR (MAP)	3, incl. sieve	no	55%	small	Wei et al. (2018)
CSTR (MAP)	2	yes	90%	0.11 mm 1	Aguado et al. (2019)
FBR (MAP)	4, vertical flow	yes	85-99%	0.3-0.6 mm	Zamora et al. (2017a)
FBR (MPP)	4, vertical flow	yes	80-90%	4 mm 2	Zhang et al. (2017)
SBR (MAP)	1	no	90-93%	-3	Etter et al. (2011), Grau et al. (2015)

1 Average value

2 Max value

3 Filtered through a cloth with a mesh width of 160 ± 50 μm

The choice of chemicals for precipitation is interesting from the aspect of costs and reactor setup. For MAP production, a large number of waste products have been employed (Aguado et al. 2019, Dai et al. 2014, Etter et al. 2011, Krähenbühl et al. 2016, Liu et al. 2013, Rubio-Rincón et al. 2014, Sakthivel et al. 2012). All those sources provided Mg^2+^ as desired, often giving rise to impurities in the struvite product, but only wood ash would influence the fertilizer product negatively due to the high content of heavy metals (Sakthivel et al. 2012). Total costs depend on the local availability of the waste resources as well as on operational costs, e.g. for precipitant dosing. An alternative to using cheap waste products is thus to lower these operational costs, e.g. by using sacrificial electrodes as done by Hug and Udert (2013) for dosing Mg^2+^, by Zheng et al. (2009) for dosing Fe^3+^, and by Zheng et al. (2010) for dosing Al^3+^.

#### 5.2.2 Adsorption processes for targeted P-recovery

For the adsorption of phosphorus, we have only found examples of anion exchange. O'Neal and Boyer (2013) and Sendrowski and Boyer (2013) tested the implementation of a commercial anion-exchange resin loaded with hydrous ferric oxide nanoparticles. Sendrowski and Boyer (2013) set up kinetic and equilibrium models for synthetic fresh and stored urine, concluding that kinetics were faster for fresh than for stored synthetic urine, but that in both cases, the maximum adsorption capacity on the resin was around 5.2 mg_PO4_·g^−1^, corresponding to 0.17 mmol_P_·g^−1^. In subsequent experiments in the same system on fresh and stored urine, O'Neal and Boyer (2013) found higher maximum adsorption capacities of 10.1 and 6.9 mg_PO4_·g_resin_^−1^, respectively, and > 92% phosphorus recovery during regeneration of the resin by a NaCl/NaOH solution.

Dox et al. (2019) investigated the use of MgAl or ZnAl layered double hydroxides (LDH) to recover phosphorus from urine by ion exchange, with the intention of direct use in agriculture as a slow-release P fertilizer. The authors obtained the best results with a MgAl LDH, with an adsorption capacity of 64 mg_P_·g_LDH_^−1^ for synthetic stored urine at pH 6, but with indications from experiments with a P-solution that this value would be around 15% lower at a realistic pH value of stored urine. This is still better than the 5-10 g mg_P_·g_ionexchanger_^−1^ cited above, but we found no explicit discussion of the suitability of aluminum-based ion-exchangers for soil conditioning. Furthermore, when it comes to weight reduction, ion exchange cannot compete with precipitation (Kabdaşlı et al. 2013). With respect to the complex production of synthetic anion exchangers, it still has to be proven that a possibly simpler operation can compensate for the disadvantage of lower volume reduction.

In some cases, we can attribute apparent adsorption to precipitation. Xu et al. (2019b) attempted to provide Al^3+^, Ca^2+^, Fe^3+^ and Mg^2+^ ions coated on biochar, but this was only successful for the dosing of Mg^2+^, most probably due to precipitation. Equally, Ganrot et al. (2008) reported apparent adsorption densities up to 10 mg_P_·g^−1^ on the cation exchanger zeolite. While Ganrot (2012) attributes this effect to anion exchange capacity of hydrous oxides of Al structural sites, Wan et al. (2017) could show that calcium ions released from zeolites lead to phosphorus precipitation in sludge reject water, with the latter explanation also fitting better to the data for urine.

### 5.3 Targeted K-recovery

A few authors suggest recovering phosphorus as MPP instead of MAP. For thermodynamic reasons, previous NH_tot_ removal is necessary to prevent the more favorable process of MAP precipitation, e.g. by stripping (Gao et al. 2018a, Huang et al. 2019) or nitrification/denitrification (Wilsenach et al. 2007). Both processes would lead to a substantial pH decrease, which is unfavorable for the process. Using MgO as magnesium source leads to a suitable pH value (Table 9), and sufficient depletion of nitrogen by stripping (section 5.1.1) would necessitate the addition of a base in the first place (Huang et al. 2019, Xu et al. 2015). Due to local difficulties of securing K fertilizer, Xu et al. (2017) and Xu et al. (2011) attempted to optimize the process for K recovery by the addition of surplus P, but despite adopting a high Mg:P ratio, concomitant high removal rates of K and P were not achieved.

## 6 Nutrient removal (only nitrogen)

Already Maurer et al. (2006) suggested biological denitrification, as well as electrochemical processes for nitrogen removal. At the time, only autotrophic denitrification had been tested, in one successful short-term experiment treating urine with sludge from a running anammox process.

### 6.1 Biological denitrification

Quantitative biological denitrification requires previous complete nitrification or nitritation. We discussed complete nitrification in section 3.1.2, but will discuss partial and complete nitritation in this section, before we proceed to the actual denitrification processes. For a general short background on nitrification and inhibition, we refer to section 3.1.

#### 6.1.1 Partial and complete nitritation

Already Maurer et al. (2006) reported that due to inhibition of nitrite oxidizers, early attempts to obtain partial nitrification in reactors with suspended solids only resulted in partial nitritation. Sun et al. (2012) observed the same effect for a sequencing batch reactor (SBR) and an MBR, not only for partial, but also in the case of complete nitrification, where others have achieved successful nitrification with suspended biomass. Operational conditions, i.e. temperature, pH and urine dilution, were similar to the ones reported in Table 2 for successful nitrification. In a similar experiment on complete nitrification, Mackey et al. (2016) achieved complete nitritation in a granular sludge SBR reactor by dosing sodium bicarbonate. The authors hypothesize that the higher pH value up to 9, as compared to earlier nitrification experiments in a similar system at pH 7.3-7.6 by Jiang et al. (2011), lead to a stronger inhibition of NOB than of ammonia oxidizing bacteria (AOB), resulting in nitrite accumulation. Pulse feeding was important for sustaining the granules, and at an inlet NH_tot_ concentration of 1500 g_N_·m^−3^, nitritation rates of up to 1100 g_N_·m^−3^·d^−1^ were obtained.

#### 6.1.2 Heterotrophic denitrification of a nitrate or nitrite solution

For urine, heterotrophic denitrification of a nitrate or nitrite solution has been suggested. The minimum COD:N ratio for complete denitrification via nitrate and nitrite is 2.86 g_COD_·g_N_^−1^ and 1.71 g_COD_·g_N_^−1^, respectively, not taking into account the COD demand for microbial growth (Udert and Jenni 2013). The COD:N ratio of around 1 in urine (Table 1) is consequently too low to support complete heterotrophic denitrification. For this reason, the process has mainly been suggested for nitrogen removal in sewers with ample provision of COD and for hydrogen sulfide control in pressure sewers. In the latter case, nitrate and nitrite can replace sulfate as electron acceptor, thereby preventing biogenic sulfide corrosion (Jiang et al. 2011, Oosterhuis and van Loosdrecht 2009).

Not surprisingly, Jiang et al. (2011) could show that heterotrophic denitrification can be obtained through the addition of nitrified urine to raw wastewater. The use of nitrite as electron acceptor may in some cases be attractive, because it requires less COD for denitrification and its toxicity inhibits the growth of sulfate-reducing bacteria and methanogens, additionally increasing the chances of total nitrogen removal through denitrification in sewers (Mackey et al. 2016). However, whereas denitrification via nitrate is a well-studied process from conventional wastewater treatment, the same process via nitrite can lead to substantial production of volatile nitrogen oxides including climate-relevant N_2_O (Schreiber et al. 2012). We have found no explicit studies on this potential detrimental effect of nitrite reduction in sewer systems, but we note that due to the fish-toxicity of nitrite, the process would only be relevant in systems without combined sewer overflows.

#### 6.1.3 Nitritation/Anammox (single and two-stage process)

Based on stoichiometry, nitritation/anammox would have a large potential for nitrogen removal from urine without additional COD (Udert et al. 2008). However, Schielke-Jenni et al. (2015) showed that in a single-stage process, nitritation/anammox and heterotrophic denitrification will coincide and their contribution to nitrogen removal cannot be differentiated with a reasonable amount of measurements. Studies by Bürgmann et al. (2011) and Huang et al. (2016) have shown that single stage nitritation/anammox is possible for NH_tot_ removal from diluted urine. Bürgmann et al. (2011) reported nitrogen removal rates of more than 430 g_N_·m^−3^·d^−1^ with an average NH_tot_ concentration in the influent of 590 g_N_·m^−3^, but for still unknown reasons the process requires exact process control in order to prevent irreversible regime shifts to a population with low anammox activity, and long-term operation was not achieved. Competition by heterotrophic bacteria alone cannot be the explanation: in experiments with sludge filtrate augmented with COD, Jenni et al. (2014) showed perfect coexistence of the anammox process and heterotrophic denitrification in concentrated solutions. Successful nitritation/anammox for undiluted urine has not been reported so far. Schielke-Jenni (2015) suggests that salt effects or inhibition by specific organic compounds are the two most likely reasons for nitritation/anammox treatment of urine being challenging.

As already reported by Maurer et al. (2006) and suggested by Chen et al. (2017), partial nitritation as discussed in section 6.1.1 could be suitable for a two-stage nitritation-anammox process. However, Schielke-Jenni et al. (2015) were unsuccessful in maintaining stable nitrogen removal in a two-stage process consisting of a Continuous flow Stirred Tank Reactor (CSTR) for nitritation and an SBR with suspended biomass for anammox. The anammox activity broke down soon after the influent was switched from digester supernatant to urine. Despite the attractive idea of removing nitrogen through nitritation/anammox in urine, the complexity seems to be high.

### 6.2 Electrochemical nitrogen removal

Electrolysis has been used to remove nitrogen compounds, i.e. urea in fresh urine and NH_tot_ in stored urine. In most of these studies, indirect oxidation with chlorine or hydroxyl radicals was the main mechanism (see section 3.3), but direct oxidation at the anode was also reported.

Zöllig et al. (2017) investigated the electrolysis of real stored urine in batch experiments with BDD and TDIROF. Besides ammonia, COD was also degraded. On BDD, ammonia oxidation was slow until all COD was degraded, but increased ten times after COD was removed (Table 10). The preferential removal of COD on BDD was probably due to breakdown of large organic molecules into small compounds by hydroxyl radicals. The small organic molecules were preferentially oxidized by chlorine. On TDIROF, ammonia and COD were degraded concomitantly from the beginning at a high rate (Table 10). Energy demand was higher for BDD (Table 10), but it must be taken into account that the experiment with TDIROF was shorter and the energy intensive phase with low ammonia and COD concentrations was not observed.

**Table 10:** Typical values for electrochemical nitrogen removal compared to denitrifying biofilm systems

Process	Degradation rate	Energy demand for oxidation	CBP	Reference
	[g_N_·m^−2^·d^−1^]	[kWh·kg_N_^−1^]	[-]	
Electrolysis on BDD 1	43-420	55	Yes	Zöllig et al. (2017)
Electrolysis on TDIROF 2	230	67	Yes	Zöllig et al. (2017)
Direct ammonia oxidation 2	2.9	42	No	Zöllig et al. (2015c)
FC 3	150	-1.8	No	
Denitrifying biofilm system	1.5-3^a^	13^b^	No	^a^Sørensen and Morgenroth (2020) ^b^Maurer et al. (2003)

1 20 mA·m^−2^, batch experiments, 100% NH_tot_ removal

2 40% NH_tot_ removal, low rates during concomitant oxidation with organics

3 See Table 11 for calculation

In experiments using TDIROF, Amstutz et al. (2012) reported a substantially lower efficiency for nitrogen removal from synthetic stored urine than from synthetic fresh urine. Ammonia electrolysis was slowed down because carbonate oxidation to percarbonate prevented the formation of chlorine, which is necessary for the indirect oxidation of ammonia. A later study (Zöllig et al. (2017) with the same type of electrodes on real stored urine, however, did not confirm these findings. The authors argued that the pH value and the carbonate concentration in real stored urine were too low to cause substantial competition by percarbonate formation.

In most studies on nitrogen removal with electrolysis, residual nitrate was formed. For example, Zöllig et al. (2017) reported that 35% of the initial ammonia in real stored urine was converted to nitrate on BDD. Amstutz et al. (2012) found that 24% of the initial urea and ammonia in synthetic fresh urine was converted to nitrate on TDIROF. Shen et al. (2019) developed a system for complete nitrogen removal: Batch experiments with real fresh urine in an EC with a photoanode and a selectively reductive Pd/Au/N-F (palladium, gold, nickel foam) cathode resulted in total nitrogen and TOC removal efficiencies of 99% and 55%, respectively.

The two main challenges of electrolytic treatment of urine at high current densities are the high energy demand (Table 10) and the production of harmful CBPs (section 3.3). Both effects are connected to the production of chlorine. Direct ammonia oxidation without mediation by chlorine was reported by Amstutz et al. (2012) for urea removal in fresh synthetic urine, and by Zöllig et al. (2015a) for ammonia removal in real stored urine. Zöllig et al. (2015a) showed that ammonia can be removed on cheap graphite when the anode potential is kept between 1.1 and 1.6 V versus a standard hydrogen electrode (SHE). In batch experiments at 1.31 V vs. SHE, an ammonia removal rate of 2.9 g_N_·m^−2^·d^−1^ and an energy demand of 42 kWh·kg_N_^−1^ was achieved (Table 10). The degradation rates were substantially lower than for indirect oxidation but in the same range as for denitrifying biofilm systems (see Table 10). It must be noted that direct ammonia oxidation can be limited by a local pH decrease at the anode, because free ammonia, but not ammonium reacts at the anode (Zöllig et al. 2015b).

One alternative to energy-consuming electrolysis could be urea degradation in fuel cells (FC, Table 10, see also section 7). The degradation rate is close to the values for indirect electrolysis and is substantially higher than for direct electrolysis or biological denitrification. Furthermore, in the electrochemical process of an FC, energy is produced and not consumed. However, only few studies exist about FC treatment of urine and little to nothing is known about possible operational challenges, if FC are operated on a long term.

## 7 Energy recovery

Energy recovery from urine was not reported by Maurer et al. (2006). Since then, FC and most notably MFC have been investigated for the direct production of electricity from urine. In FC applications, urea from fresh urine is oxidized at the anode, while in MFC the electron donors are organic substances with a chemical oxygen demand, i.e. substances, which contain reduced carbon. MFC can be fed with fresh or stored urine. The maximum power production in FC and MFC is below 1 W·p^−1^ (Table 11) and therefore too low to substantially contribute to the electricity demand of an industrialized country. For comparison, the average electric power consumption in Switzerland was 760 W·p^−1^ in 2019 (BFE 2020). Also the achievable power densities per anode surface are low. Lan and Tao (2011) reported a maximum of 11 W·m^−2^ for FC at 20°C with fresh urine, while Barbosa et al. (2017) reported 0.95 W·m^−2^ for MFC, with stored urine. These values were measured in short-term polarization experiments. In long-term MFC experiments, the power densities are even lower, with reported power densities of 250 mW·m^−2^ (Kuntke et al. 2012), 311 mW·m^−2^ (Zhou et al. 2015) and 314 mW·m^−2^ (Barbosa et al. 2017). For FC, no continuous long-term experiments have been reported. All reported power densities for FC and MFC are substantially lower than the power densities, which can be achieved in hydrogen FC: Xu et al. (2016a) cited values of 5000 to 6000 W·m^−2^ for proton exchange membrane FC operated with hydrogen.

**Table 11:** Typical examples for fuel cell (FC) and microbial fuel cell (MFC) performance with urine

Process	Electron donor			Polarization		Long-term		Own calculations		Reference
	Reductant	Load	Degradation	Power density 1	Voltage	Coulombic efficiency 1	Average voltage 2	Power production	Degradation rate	
		[gp^−1^d^−1^]	[%]	[W·m^−2^]	[V]	[-]	[V]	[W·p^−1^]	[g·m^−2^·d^−1^]	
FC 3	Urea-N	11.5	90	11	0.32	98	-	0.78	150	Lan and Tao (2011)
MFC 4	COD	12.5	75.5	0.95	0.46	27	0.62	0.22	41	Barbosa et al. (2017)

1 Maximal values

2 Corresponds to maximal Coulombic efficiency

3 Short term polarization measurements with fresh urine at 20°C. Degradation is assumed and Coulombic efficiency is from Xu et al. (2014), urea load from Udert et al. (2006). Power production was calculated with voltage from polarization experiment

4 Short-term polarization measurements and long-term experiments (about 120 d) with stored urine after phosphate precipitation. Electrochemical performance data are average values from two experiments.

The amount of energy, which can be recovered from a substrate depends on the extent of substrate degradation and on the Coulombic efficiency. A typical Coulombic efficiency for MFC is around 25% (see e.g. Barbosa et al. (2017)), but values vary widely. Ieropoulos et al. (2012) reported values between 22 and 70% and Santoro et al. (2014) reported only 2.1% with urine, while the COD removal efficiency was 85%. Possible reasons for low Coulombic efficiencies are the competition with other COD-consuming processes such as sulfate reduction (Santoro et al. 2013), methanogenesis (Barbosa et al. 2017) or the consumption of oxygen, which diffuses from the cathode into the anode chamber (Liu et al. 2004). In contrast to MFC, Coulombic efficiencies in FC can be very high. Xu et al. (2016b) reported a Coulombic efficiency of 98% when using a Cr(VI) solution as catholyte. However, we did not find any other values for the Coulombic efficiency in urine FC.

In addition to the low power production, the material costs are an additional challenge for electricity production. Material costs can be particularly high for anodes used in FC. To reduce costs, Lan et al. (2010) studied the use of nickel instead of platinum as anode material. Lan and Tao (2011) and Xu et al. (2014) aimed at increasing the performance of nickel anodes by using nano-sized nickel or nickel alloys with cobalt, respectively. While anodes of MFC usually consist of cheap graphite, the focus of reducing material costs for MFC has been mainly on CEM. Instead of engineered CEM, ceramic membranes (Pasternak et al. 2016), natural rubber gloves (Winfield et al. 2014) and different types of paper (Winfield et al. 2015) were tested. However, the most promising setups are single chamber MFC with membrane-less air cathodes (e.g., Santorio et al. (2013)).

Since urine-based FC and MFC cannot contribute substantially to the overall electricity consumption of a society, researchers explored special applications, e.g small or mobile devices such as mobile phone chargers (Ieropoulos et al. 2013b), heartbeat actuators (Walters et al. 2013), emergency location transmitters (Winfield et al. 2015) or wireless transmitters (Taghavi et al. 2015). In practical applications, stacks of small MFC are used to increase power densities and to prevent cell polarity reversal (Ieropoulos et al. 2013a). Additionally, the stacks are arranged in series (see, e.g., Ieropoulos et al. (2013b)) to increase the low voltage of single MFC (see Table 11). While electricity production from urine with MFC and FC is probably only interesting for some niche applications, these systems could provide fast and energy-efficient COD or nitrogen removal, respectively (see sections 3.3 and 6.2).

In some studies on electrochemical urine treatment, energy-rich hydrogen is produced at the cathode. Kim et al. (2013) and Luther et al. (2015) calculated energy savings of 10% and 36%, if hydrogen was used to recover electricity in a fuel cell.

Christiaens et al. (2017) produced protein with the help of hydrogen oxidizing bacteria. A totally different approach of energy recovery was used by Mercer et al. (2019). The authors showed that after water removal by MD, part of the thermal energy could be recovered as electrical energy with reverse ED. The authors suggest that such a system could be used to convert waste heat used for MD to electricity in order to support the electricity requirements of off-grid sanitation systems in low-income countries.

## 8 Removal of pathogens and organic micropollutants

### 8.1 Removal of pathogens

Although urine is normally free of pathogens as long as it is contained in the human bladder, fecal contamination often occurs during urine collection in urine-diverting toilets or urinals. Consequently, storage to obtain sufficient sanitization for direct use of urine as fertilizer was one of the first processes reported for source-separated urine (Maurer et al. 2006). In view of the importance, surprisingly little effort has been invested in the methods for sanitization since then. Except for one – unsuccessful – example of UltraViolet radiation (UV) inactivation of pathogens in urine (Giannakis et al. 2018), pathogen inactivation or separation from the nutrients has mainly been reported, or can be assumed, as a side-effect of a main urine treatment process. Distillation at temperatures beyond 55°C lead to heat inactivation and during nitrogen stripping, we expect the non-volatile pathogens to remain in the solution. For alkaline drying, Senecal et al. (2018) investigated the fate of pathogens after dehydration of urine in wood ash. All bacteria and phages were removed to below the detection limit within four days of storage at 20°C, but the nematode *Ascaris suum* required substantial time in order to obtain a log 3 reduction (325 days at 20°C and 9.2 days at 42°C). However, the expected concentration of nematodes in the resulting fertilizer product would already be below the WHO guidelines for unrestricted use due to the limited fecal contamination of urine. In many other cases, we must expect drying combined with short heat treatments to be the most obvious method of sanitization. This would only be counter-indicated in the case of struvite, which decomposes at temperatures above 55°C (Bhuiyan et al. 2008), giving rise to ammonia losses. Decrey et al. (2011) and Bischel et al. (2016) investigated the inactivation of virus phages, helminth eggs (*Ascaris suum*), heterotrophic and total bacteria, as well as *Enterococcus spp.* and *Salmonella typhimurium* during drying of struvite, all at 5–35°C and at a relative humidity of 40 – 80%. Higher temperatures and decreased moisture increased deactivation, but it was concluded that a short heat treatment above 55°C before drying would be required for the struvite product from urine to be safe for use in agriculture. Bischel et al. (2015b) investigated the fate of viral and bacterial pathogen surrogates during urine nitrification. They found that some inactivation occurred but they concluded that nitrification was insufficient as a stand-alone technology for sanitization of source-separated urine.

### 8.2 Removal of organic micropollutants

From conventional WWTPs, it is known that while biological treatment alone will not lead to sufficient removal, activated carbon is effective for unspecific removal of organic micropollutants from treated wastewater, albeit with strong competition from dissolved organic matter (Benstoem et al. 2017). For urine, it may thus be most economic to use the method after biological treatment (see section 3.1), but up to 2019, we have found no scientific articles on this topic. In practice, however, the effective removal of organic micropollutants from biologically treated urine by activated carbon has led to the licensing of the urine-based fertilizer Aurin for all crops in Switzerland (www.vuna.ch). Ozonation is another well-established process for unspecific removal of micropollutants in wastewater, again in strong competition with the oxidation of organic matter (Bourgin et al. 2018). The process functions for urine too, but with a much higher energy demand than for wastewater treatment and a high production of toxic by-products (Dodd et al. 2008, Fitzke and Geißen 2007, Gajurel et al. 2007, Gulyas et al. 2007, Tettenborn et al. 2007). There has been no follow-up publications on the topic since 2008.

For urine, there are two different main goals when dealing with organic micropollutants: separation from nutrients and removal. With a focus on fertilizer production, one may consider the separation of nutrients and organic micropollutants sufficient. This effect has mainly been shown for precipitation of struvite, where Ronteltap et al. (2007a) and Wei et al. (2018) observed consistent low inclusion, with 95-99% of the spiked organic micropollutants remaining in the liquid. Other authors reported inclusion only before washing (de Boer et al. 2018) or without indicating any washing of the struvite crystals (Schürmann et al. 2012).

In fresh urine, nano filtration (NF) has been tested for separation of nutrients from micropollutants. Lazarova and Spendlingwimmer (2008) report close to 90% retention of the micropollutants, but only 40% permeation of urea. This is in contrast to previous results on NF, where Pronk et al. (2006b) found excellent permeation of urea. In addition, Cristóvão et al. (2019) studied the separation of anticancer drugs from synthetic fresh urine by NF, with more than 89% rejection by the best NF material, albeit with close to 20% co-rejection of urea and monovalent ions, and 32-99% for multivalent ions.

With a focus on water pollution control, there is a large opportunity for removal of organic micropollutants from urine and not only for separation (see section 1 for a discussion). For urine with low COD content, activated carbon has already proven highly effective in practice. For urine with a high content of COD, however, there is a considerable research gap, with only little information available. Whereas many authors have performed experiments on advanced oxidation processes (AOPs) and adsorption with the purpose of removing one or several pharmaceuticals, the majority conducted the experiments with synthetic urine containing no or little COD. We therefore only give a short summary of the literature on these processes in order to show where some process information is available as a basis to conduct further experiments with real urine containing realistic amounts of COD.

We note that after 2019, there has been a long required boost of publications on the removal of organic micropollutants, which we unfortunately did not catch in this review.

#### 8.2.1 Advanced oxidation processes (AOPs)

Low-pressure UV, alone or in combination with hydrogen peroxide (H_2_O_2_), peroxydisulfate (PDS) and H_2_O_2_/Fenton, has been tested on different drugs in synthetic urine, with the combinations with PDS and H_2_O_2_/Fenton reaching the best results (Giannakis et al. 2017a, Giannakis et al. 2017b, Giannakis et al. 2017c, Zhang et al. 2015, Zhang et al. 2016a, Zhang et al. 2016b). Further, Luo et al. (2019) tested ferrate (FeO_4_^2−^) and Wang et al. (2019) biochar-activated monochloramine for the oxidation of selected pharmaceuticals, both with good results in the selected settings.

#### 8.2.2 Adsorption processes for removal of organic micropollutants

Adsorption has not only been suggested for removal of organic micropollutants, but also for targeted recovery of nutrients (sections 5.1.2 and 5.2.2). Only few authors, however, have questioned whether co-adsorption of nutrients and organic micropollutants would play a role. Tarpeh et al. (2017), for instance, proposed post-treatment for removal of organic micropollutants after nutrient adsorption. Only Sendrowski and Boyer (2013) tested co-adsorption experimentally and in fact found high co-removal of the pharmaceutical diclofenac in an anion exchange process for P-recovery.

A number of authors have studied the adsorption of specific pharmaceuticals on adsorbents of different origin, mostly biochars (Paredes-Laverde et al. 2019, Paredes-Laverde et al. 2018, Solanki and Boyer 2017, Sun et al. 2018) and strong base anion exchange resin (Landry and Boyer 2013, Landry et al. 2015). The authors mostly found no significant differences between fresh and stored synthetic urine. Only few of the authors addressed the two major concerns for adsorption of organic micropollutants: competition from dissolved organic matter and possible co-adsorption of nutrients. Solanki and Boyer (2019) found significant lower adsorption on biochar for real than for synthetic urine, but again with no differences between fresh and stored urine. Based on batch experiments, the authors suggest to use the double amount of biochar than for synthetic urine, i.e. 40 g_biochar_·L^−1^, corresponding to 60 g_biochar_·p^−1^·d^−1^. As seen in section 5.1.2, biochar is used for targeted nitrogen removal in fresh and stored urine at high pH values, leading to excellent results. We would therefore expect substantial co-adsorption of nitrogen in both types of urine. In fact Solanki and Boyer (2017) observed exactly this, with the problem being especially pronounced for activated carbon with 20% N-removal and a little more surprisingly also 40% P-removal. While it was difficult to prove the actual removal mechanisms, the authors argue that in both cases, co-adsorption would be a plausible explanation. In municipal wastewater treatment, however, adsorption of ammonia or phosphate on activated carbon is commonly not observed.

#### 8.2.3 Pharmaceutical removal in electrochemical processes

In a few studies, ECs were used to remove pharmaceuticals either by oxidation or reduction. On the one hand, indirect oxidation on BDD (Cotillas et al. 2018) and Ti-IrO_2_ anodes (Jojoa-Sierra et al. 2017) was shown to be a suitable process for antibiotics removal. Pharmaceuticals and intermediates could be removed completely, though degradation of organic compounds slowed down their oxidation. On the other hand, De Gusseme et al. (2011) investigated the reductive dehalogenation of the iodinated X-ray contrast medium diatrizoate in an MEC with a cathode containing biogenic palladium nanoparticles.

ED was tested on the removal of pharmaceuticals with membranes. Pronk et al. (2007) reported that during a one-year long pilot experiment with real stored urine, diclofenac, carbamazepine and propranol were removed below the detection limit, while ibuprofen was removed to a large extent. In addition, estrogenic activity was reduced by 90%.

## 9 Qualitative comparison of technologies

The technology overview in Table 12 shows that many technologies are well understood with respect to the recovery or removal of nutrients, water and COD. The main knowledge gaps exist for pathogen and micropollutant removal. For some processes, a certain performance during urine treatment can be assumed based on known properties of the technologies. We presumed that membranes used for nanofiltration, membrane distillation, and cation and anion exchange provide a good removal of pathogens and pharmaceuticals based on studies by Pronk et al. (2006) for nanofiltration and Pronk et al. (2007) for ED. From conventional wastewater treatment, it is known that processes like ozonation and AOP are well suited for pathogen removal. During distillation, urine is heated to approximately 80°C, which is above the requirements for pasteurization. For all biological processes, we presumed no substantial effect on pathogen removal based on the study by Bischel et al. (2015) for urine nitrification.

**Table 12:** Qualitative comparison of technologies discussed in this review (for assumptions, please see text)

	N recovery	P recovery	K recovery	Water removal	N removal	Stabilization: COD removal	Stabilization: NH_3_ loss	Pathogen removal	Micropollutant removal	Energy production	Technology readiness
*Stabilization*											
Partial nitrification	o	o	o	o	o	++	++	o	TBD	o	++
Complete nitrification	o	o	o	o	o	++	++	o	TBD	o	++
Acid dosage	o	o	o	o	o	o	++	+	TBD	o	+
Base dosage	o	++	o	o	o	o	++	++	TBD	o	++
Electrolysis, indirect oxidation	o	o	o	o	++	++	o	++	+	o	+
Microbial fuel cell	o	o	o	o	o	++	+	+	TBD	+	+
*Volume reduction*											
Drying	++	++	++	++	o	o	o	++	TBD	o	++
Distillation	++	++	++	++	o	o	o	++ ⁎	TBD	o	++
Forward osmosis	++	++	++	++	o	o	o	++ ⁎	++ ⁎	o	o
Membrane distillation	+	++	++	++	o	o	o	++ ⁎	++ ⁎	o	o
Electrodialysis	+	+	+	+	o	+	o	++ ⁎	++	o	++
Algae growth	++	++	++	+	o	o	++	o ⁎	TBD	o	o
*Targeted nutrient recovery*											
Ammonia stripping	++	o	o	o	o	o	++	o	o	o	++
Ammonium adsorption	++	o	o	o	o	o	++	TBD	TBD	o	+
Ammonium concentration with ED	++	o	o	o	o	++	++	++ ⁎	TBD	o	+
Phosphate precipitation with Mg	o	++	o	o	o	o	o	TBD	+	o	++
Phosphate precipitation with Fe/Al	o	++	o	o	o	o	o	TBD	TBD	o	++
Phosphate precipitation with Ca	o	++	o	o	o	o	o	++	TBD	o	++
Phosphate adsorption	o	++	o	o	o	o	o	TBD	TBD	o	o
Potassium precipitation	o	+	+	o	o	o	o	TBD	TBD	o	o
*Nutrient removal*											
Heterotrophic denitrification	o	o	o	o	++	++	++	o ⁎	TBD	o	+
Nitritation/anammox	o	o	o	o	++	++	++	o ⁎	TBD	o	o
Electrolysis, indirect oxidation	o	o	o	o	++	++	o	++	+	o	+
Direct ammonia electrolysis	o	o	o	o	++	o	o	TBD	TBD	o	o
Fuel cell	o	o	o	o	++	o	++	TBD	TBD	+	o
*Energy recovery*											
Fuel cell	o	o	o	o	++	o	++	TBD	TBD	+	o
Microbial fuel cell	o	o	o	o	o	++	+	+	TBD	+	+
*Pathogen removal*											
UV	o	o	o	o	o	o	o	++	TBD	o	o
Heating	o	o	o	o/+	o	o	o	++	TBD	o	o
*Removal of organic micropollutants*											
Ozonation	o	o	o	o	o	+	o	++ ⁎	++	o	+
Nanofiltration	o	o	o	o	o	o	o	++ ⁎	++	o	+
Advanced oxidation processes	o	o	o	o	o	+	o	++ ⁎	++	o	o
Adsorption	+	+	o	o	o	o	o	TBD	++	o	+
Electrolysis, anodic indirect oxidation	o	o	o	o	++	++	o	++	+	o	+
Electrolysis, cathodic reduction	o	o	o	o	o	o	o	TBD	+	o	+

o No effect.

+ Some positive effect.

++ Strong positive effect.

TBD To be determined, effect unclear.

⁎ Presumed based on general technological properties, but not shown for urine treatment.

A quantitative assessment of the suitability of the technologies was not the goal of this paper. However, this review can serve as a first step towards such an assessment, which should also include the resource demand of the different technologies. Besides energy and chemical consumables, the resource demand should also include installation and maintenance costs for reactors as well as space requirements. However, for most technologies, much of this information is not available yet or not reliable due to the low technology readiness level.

## 10 Conclusions

Our review for the years 2006-2019 has shown that the research community has made tremendous progress in the development of urine treatment technologies. No industrialized urine treatment reactors exist to date for any of the processes, but many technologies are close to industrial optimization. Based on our review we see the following trends for the further development for urine treatment technologies:

• Urine treatment should be as close as possible to the source to prevent transport costs. Most technologies were therefore developed for reactors at bathroom scale or at the scale of larger building or building complexes.
• Physical-chemical processes, especially alkaline stabilization combined with evaporation, adsorption or membrane separation processes seem to be particular suitable at bath room scale. Challenges are the requirements for reagents, energy and maintenance, such as membrane cleansing.
• Biological processes are arguably the most efficient technologies for urine stabilization, but due to possible inhibition effects by salt or free ammonia, they are more robust at larger scale.
• Efficient processes for water removal, such as distillation, or for nutrient recovery, such as ammonia stripping and phosphate precipitation are also better suited for large scale application due to their technical complexity.
• A growing new field of technologies involve electrochemical processes. They provide new possibilities such as electricity generation or on-site disinfection, but challenges such as low efficiencies and high process complexities still need to be overcome.
• Most technologies developed so far focus on nutrient recovery or removal. More development is needed for the removal of pathogens and micropollutants. However, it should be noted that since 2019, important improvements have been made, which were not included in this review. Another topic to be investigated in more detail is the treatment or disposal of side streams, especially in the case of single nutrient recovery processes.

## Declaration of Competing Interest

The authors declare that they have no known competing financial interests or personal relationships that could have appeared to influence the work reported in this paper. The authors declare the following financial interests/personal relationships, which may be considered as potential competing interests: Kai M. Udert is co-owner of the Eawag spin-off Vuna Ltd. The company uses biological and physical processes for nutrient recovery from urine. The study was not influenced by the relationship of Kai M. Udert with Vuna Ltd.
